# Kinetic vitrification: concepts and perspectives in animal sperm cryopreservation

**DOI:** 10.1590/1984-3143-AR2022-0096

**Published:** 2023-05-22

**Authors:** Bianca Barreto Barbosa, Inara Tayná Alves Evangelista, Airton Renan Bastos Soares, Danuza Leite Leão, Ricardo José Garcia Pereira, Sheyla Farhayldes Souza Domingues

**Affiliations:** 1 Laboratório de Biotecnologia e Medicina de Animais da Amazônia, Universidade Federal do Pará, Castanhal, PA, Brasil.; 2 Programa de Pós-graduação em Saúde e Produção Animal na Amazônia, Universidade Federal Rural da Amazônia, Belém, PA, Brasil.; 3 Instituto de Desenvolvimento Sustentável Mamirauá, Tefé, AM, Brasil.; 4 Departamento de Reprodução Animal, Faculdade de Medicina Veterinária e Zootecnia, Universidade de São Paulo, São Paulo, SP, Brasil.

**Keywords:** assisted reproduction, cryobank, cryopreservation, cryoprotectant-free, spermatozoa

## Abstract

Sperm cryopreservation is an important tool for genetic diversity management programs and the conservation of endangered breeds and species. The most widely used method of sperm conservation is slow freezing, however, during the process, sperm cells suffer from cryoinjury, which reduces their viability and fertility rates. One of the alternatives to slow freezing is vitrification, that consist on rapid freezing, in which viable cells undergo glass-like solidification. This technology requires large concentrations of permeable cryoprotectants (P- CPA’s) which increase the viscosity of the medium to prevent intracellular ice formation during cooling and warming, obtaining successful results in vitrification of oocytes and embryos. Unfortunately, this technology failed when applied to vitrification of sperm due to its higher sensitivity to increasing concentrations of P-CPAs. Alternatively, a technique termed ‘kinetic sperm vitrification’ has been used and consists in a technique of permeant cryoprotectant-free cryopreservation by direct plunging of a sperm suspension into liquid nitrogen. Some of the advantages of kinetic vitrification are the speed of execution and no rate-controlled equipment required. This technique has been used successfully and with better results for motility in human (50-70% motility recovery), dog (42%), fish (82%) and donkey (21.7%). However, more studies are required to improve sperm viability after devitrification, especially when it comes to motility recovery. The objective of this review is to present the principles of kinetic vitrification, the main findings in the literature, and the perspectives for the utilization of this technique as a cryopreservation method.

## Introduction

Cryopreservation or biostabilization is a stable and long-term preservation and storage method in which biological materials such as cells, tissues, and embryos are kept in a glassy state (Katkov et al., 2012) and do not undergo biological changes (Akiyama et al., 2019). Although sperm cryopreservation has undergone some changes over the years, conventional freezing (fast or slow) is still the most used method to cryopreserve human and animal sperm samples (Isachenko et al., 2017). This freezing method requires specialized equipment, takes time, and often damages sperm (Ozkavukcu et al., 2008), mainly owing to the reduced thawing temperature, formation of ice crystals, and stress (physical, chemical, osmotic, and oxidative) that compromise sperm quality and fertilizing capacity (Colás et al., 2009). The main causes of reduced sperm quality in slow freezing are changes in the lipid phase and/or the increase in lipid peroxidation, which results in a reduction in the speed and percentage of mobile sperm and significant losses in the fertilization potential (Celeghini et al., 2008).

To prevent damage, most cryopreservation methods, including slow freezing, use P-CPA that can move across cell membranes and modulate the rate and duration of cell dehydration during membrane phase transitions induced by freezing. P-CPA provide intracellular protection because, among other reasons, they reduce the temperature of ice nucleation and the size of crystals formed (Swain and Smith, 2010; Sieme et al., 2016).

Equilibrium vitrification, which is used for human (Valojerdi et al., 2009) and animals (Varago et al., 2006; Gibbons et al., 2019) embryo preservation, requires high concentrations of cryoprotectant; this raises the viscosity of the milieu and prevents ice formation during cooling, as well as during warming. However, high concentrations of cryoprotectant are harmful to the cytoskeletons of oocytes, and especially to those of sperm cells due to harmful cytotoxic (Fahy, 1986; Pegg, 2015) and osmotic (Guthrie et al., 2002; Glazar et al., 2009) effects. In this context, this technique is not recommended for sperm cells, owing to the use of high concentrations of these cryoprotectants, which can compromise the cells’ biological function (Mphaphathi et al., 2012). Exceptions include the results obtained by Consuegra et al. (2019), who used equilibrium vitrification without applying P-CPA to equine sperm. Thus, cryobiologists are increasingly demanding options to reduce the deleterious effects of cryoprotectants that permeate sperm cells; such measures suggest a reduction (Thananurak et al., 2019) in the frequency of cryoprotectant use or their removal from cryopreservation protocols.

From this perspective, an alternative to the use of P-CPA is kinetic vitrification (Katkov et al., 2012), in which permeable cryoprotectants are not used and the use of N-PCA is optional. Thus, driven by the results of studies using this method in human sperm (Isachenko et al., 2004a, b, 2008, 2012), researchers have been evaluating and comparing seminal quality in animal models, such as fish (Merino et al., 2011) and rabbits (Rosato and Iaffaldano, 2013). In such scenarios, kinetic vitrification is an alternative to equilibrium or conventional vitrification methods for sperm cells, and stands out for its fast execution (Kim et al., 2012), not requiring the use of P-CPA, and not requiring programmable refrigeration devices (Ozkavukcu and Erdemli, 2002), thereby being a more cost-effective method (Tao et al., 2020). Thus, this review aimed at presenting the principles of kinetic vitrification, its application in sperm samples, the main findings in the literature, and perspectives for the use of this cryopreservation method. Compiling primary studies on this subject is essential to help researchers make decisions about the application of this methodology to different experimental models.

## Sperm cell cryopreservation: methods and concepts

**Figure 1:**
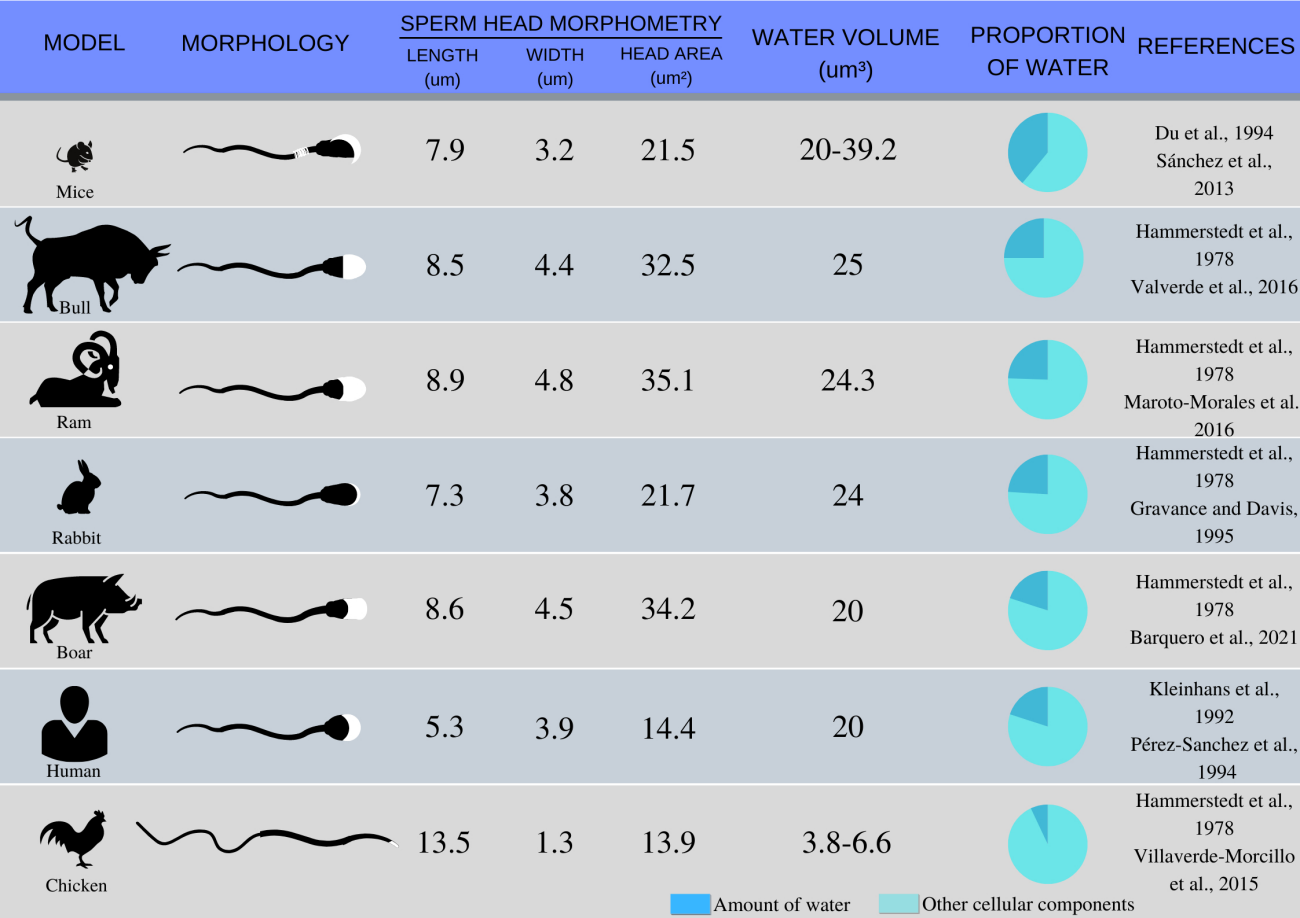
Water proportion graph based on spermatozoa water volume and sperm head morphometry.

Biological and chemical reactions in living cells are dramatically reduced at low subzero temperatures, a phenomenon that can lead to the possible long-term preservation of cells and tissues (Jang et al., 2017; Yashaswi and Mona, 2022). Biopreservation begins with a reduction in temperature from 37 °C to the 0-10 °C range and cryopreservation at -196 °C is considered an effective method for sperm preservation, which can maintain its structural and functional integrity after thawing (Li et al., 2019; Nagata et al., 2019; Silva et al., 2019). Water plays a central role in cryobiology. A cell consists of around 60 to 85% water both in free and bounded forms (Yashaswi and Mona, 2022) and when refer to sperm cell, different content of water can occur (Hammerstedt et al., 1978; Kleinhans et al, 1992; Du et al., 1994; Pérez-Sánchez et al., 1994; Gravance and Davis, 1995; Maroto-Morales et al., 2010; Sánchez et al., 2013; Villaverde-Morcillo et al., 2015; Valverde et al., 2016; Barquero et al., 2021) as show in Figure 1. The bound form refers to the water hydrated to complex mixtures of cells like proteins, lipids and glass transition temperature of pure water and no detectable biochemical activity is possible due to lack of sufficient thermal energy. Moreover, the progressive reduction and ultimately the absence of liquid water (once completely frozen) limit all metabolic processes (Pegg, 2007).

This loss of kinetic energy of molecules results in the uncoupling and re-coupling (shunting) of biochemical reactions (Tani and Neely, 1989). In addition to metabolic imbalances measurable changes in cell and organelle membrane lipid domains occur. These structural characteristics (transitions) result in a change in membrane fluidity from the liquid-crystalline state to the solid gel state yielding a “leaky” membranous state (Baust et al., 2009). Freezing is removal of water so that it transforms the liquid water into ice either when within the cell or after it flows out of the cell and freezes externally. The major hurdle for cells to overcome at low temperatures is the water to-ice phase transition (Jang et al 2017). Ice crystal formation requires at least one initial nucleation event. The rate of nucleation of intracellular ice crystals is a function of temperature and cytoplasm composition. In classical nucleation theory, a stable ice nucleus is formed by random clustering of water molecules (Karlsson et al., 1993). Hence, the two kinetic processes occurring during the cooling of cells that is growth of ice and the loss of water from the cell, happens at a characteristic rate. This is highly influenced by the cooling rate imposed on the system (Mazur, 2004).

In slow freezing, cells are cooled in suspension and ice nucleates first in the extracellular space (Hagiwara et al., 2009) leading to the biophysical responses of cellular dehydration. In case a controlled reduction of temperature is maintained, a sufficient osmotic pressure persists that prevents the formation of ice crystals within the cell. However, the cells continually shrink during the process due to the efflux of water (Pegg, 2015). During rapid freezing, there is less time for water to move in the extracellular compartment and gets supercooled very fact leading to intracellular ice formation.

In vitrification the property that viscous liquids allow rapid cooling far below their melting temperature and undergo solidification by avoiding crystallization is used. The supercooled substance with the physical properties of a liquid subsequently acquires solid properties once it reaches below a particular temperature called the glass transition temperature (Tg) (Fahy and Wowk, 2015). At this point, the molecules of the substance remain in a disordered pattern as in liquids but are locked in place and the consequent “solid-liquid” is called as the glass (Wowk, 2010). Since there is no crystallization event, vitrification outruns the processes of ice nucleation and growth and thus their potential adverse effects.

**Figure 2:**
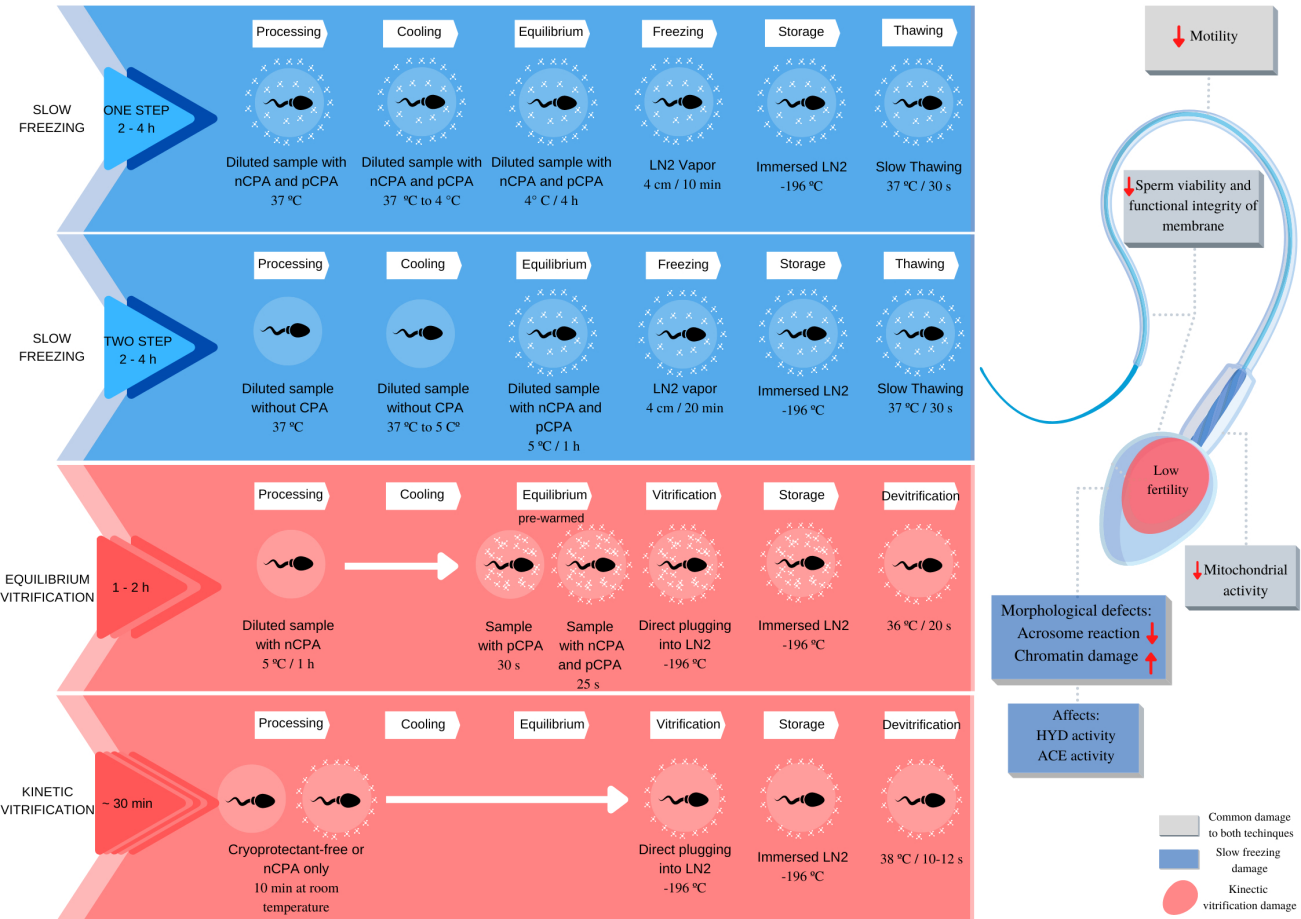
Main sperm cryopreservation protocols. Slow freezing protocol-one step (Khalil et al., 2018); slow freezing protocol-two step (Brito et al., 2017); equilibrium (Baiee et al., 2020) and kinetic vitrification protocols (Rosato and Iaffaldano, 2013).

Currently, two main approaches for sperm cryopreservation are used on cells, organs, and tissues: conventional freezing (slow and fast) and vitrification (equilibrium and kinetics). However, several methodologies have been used to preserve the fertilizing capacity of sperm cells, differing in terms of the devices used, dilution rates, composition of cryoprotective agents (CPA), freezing rates, and thawing protocols (Li et al., 2019). Furthermore, slow freezing can be divided into different protocols (Figure 2) such as the One-step that consists of diluting the sample with cryoprotectants and the Two-step in which cryoprotectants are added after cooling (equilibration). And vitrification is divided into equilibrium or traditional and kinetic vitrification (Katkov et al., 2012).

In conventional slow freezing, the sample is gradually cooled from room temperature to 4– 5 °C at a rate of 0.5-1 °C/min. Then, the temperature is further reduced to -80 °C at a rate of 1–10 °C/min and the sample is immersed in liquid nitrogen at -196 °C (Thachil and Jewett, 1981). The reduction in temperature is associated with low concentrations of P-CPA and N-CPA (Li et al., 2019). However, this method takes time, and can last 2–4 h, which varies according to the target species and requires the use of a freezer (Silva et al., 2019; Zainuddin et al., 2020) or a programmable freezer (Santo et al., 2012). Conversely, in the conventional rapid freezing protocol, after the addition of a cryoprotectant, the sample comes into direct contact with nitrogen vapors at -80 °C at 15-20 cm for 15 min and only then the sample is immersed in liquid nitrogen (-196 °C) (Sherman, 1990). Fast protocols differ from slow protocols in that (i) much of the dehydration and cryoprotective permeation occurs before the start of cooling, and (ii) cooling is usually performed in a single step, in which the sample is directly cooled from a temperature > 0 °C to sub-zero temperatures (< 130 °C) (Shaw and Jones, 2003). Thus, to optimize time and cost in the sperm cryopreservation process in humans, researchers have focused on the cryopreservation of sperm cells through vitrification, which can be performed with an equilibrium vitrification protocol or with a method that does not use P-CPA, known as kinetic vitrification (Li et al., 2019).

The vitrification process is defined as a method to solidify the liquid at low temperatures, in an amorphous or glassy state, without the formation of extra and intracellular ice crystals in the cryopreserved cells and tissues during the process and during the devitrification of recovered biological material (Kuleshova and Lopata, 2002). It is the combination of thermodynamic and kinetic effects that allow ice crystal nucleation and growth to be avoided during cooling of these solutions and is the means by which vitrification of oocytes and embryos are achieved. However, if the system is afforded sufficient time during warming,i.e., if the heating rate is slow, the molecules in the ice crystals may rearrange to form the more favorable hexagonal crystal structure as well as larger crystals (Schiewe and Mullen, 2018). According to MacFarlane (1986) this structure of ice is the most damaging to biological systems. This approach was first proposed in 1937 by Swiss priest Basile J. Luyet, who is considered the founder of cryobiology. Although his theory was tested in a practical application in organic liquids, the cooling rate at the time could not meet the vitrification requirements (amorphous/glassy state). For Luyet, “[...] the essential problem of the vitrification technique is... to obtain a cooling speed sufficient to prevent the formation of crystals” (Luyet, 1937a, b).

However, the use of P-CPA and/or drying proposed by Luyet et al. was not initially intended to decrease the rate of crystallization or decrease the maximum amount of water crystallization possible, but to dehydrate the biological sample immediately before cooling to minimize the amount of water required in the process (Luyet, 1937b). As vitrification requires very high cooling rates, Luyet et al. conducted studies using rapid cooling techniques between 1937 and 1958 (Luyet and Rapatz, 1958). These years are considered the era of rapid cooling with little or no intracellular cryoprotection, as the use of cryoprotectants to minimize frostbite injuries was not known until 1949 (Fahy, 2015), when Polge et al. (1949) made a crucial discovery, that the use of glycerol (a permeable solute) could provide protection to cells at low temperatures.

The first report of cryopreservation by vitrification in sperm consisted of an experiment with frogs by Luyet and Hoddap, in 1938, in their study entitled “*Revival of frog's spermatozoa vitrified in liquid air*” (Luyet and Hodapp, 1938). The vitrification method used by these authors became known as kinetic vitrification, which was characterized by its ultra-fast cooling (tens of thousands of °C/min) without P-CPA (Luyet and Hodapp, 1938; Luyet, 1937a, b). In later years, studies conducted in rabbit kidneys by Greg Fahy et al. using high pressure and extremely high concentrations of P-CPA, inaugurated another vitrification method that became widely known among cryobiologists as the equilibrium vitrification (Fahy et al., 1984). Since then, it was established that vitrification could only be achieved using high concentrations of combinations of P-CPA and N-CPA (Fahy, 1986). However, the toxicity of these physicochemical agents related to osmotic damage during saturation, as well as biochemical changes in the sperm cell, have been described as limiting factors for cryobiology by equilibrium vitrification (Mazur et al., 2000).

Thus, to avoid these toxic effects, some researchers have questioned whether P-CPA are necessary for successful vitrification, proposing the use of very fast heating and cooling rates (50,000 K/min or more) in a very small sample size (Nawroth et al., 2002), without using P-CPA and using only sucrose and other agents such as N-CPA, thus recovering kinetic vitrification, also known as “ultra-fast freezing” (Isachenko et al., 2004a, 2008). This methodology is an alternative to the conventional equilibrium sperm vitrification, owing to the low tolerance to sperm osmotic changes from mammals and birds (Donoghue and Wishart, 2000), which can be caused by high concentrations of P-CPA (Isachenko et al., 2003).

Regarding the differences between cellular damage caused by the cryopreservation process including cooling rates and the use of solutions with or without cryoprotectants, Fig 2 shows that sperm motility, viability, and mitochondrial activity are common cellular damages associated with cryopreservation techniques. Slow freezing can cause morphological damage, reduced acrosomal reaction, and increased chromatin damage. There is also a reduction in the activity of sperm acrosome enzyme (ACE) and hyaluronidase enzyme (HYD) that are positively correlated with motility (Sun et al., 2020) The cellular damage caused by kinetic vitrification refers mainly to loss of motility and consequently fertility.

## Essential factors for vitreous state

Three factors are reported to be essential for obtaining the glassy state: increase cooling rate, increase viscosity and decreasing sample volume (Arav et al., 2002).

Cryopreservation methods vary according to freezing speed (exposure of the sperm solution to low temperatures per minute), cryoprotectant concentration and temperature reduction rates, which includes slow freezing (0.5-0 °C/minute), rapid freezing (50-400°C/minute), ultrarapid freezing (approximately 2500 °C/minute), and vitrification (approximately 20 000 °C/minute) (Schulz et al., 2020).

In this context, the addition of P-CPA has become essential in conventional cryopreservation methods i.e. slow freezing for cells and equilibrium vitrification for organs and tissues, to protect the sperm cell from damage caused by exposure to low temperatures (Medeiros et al., 2002). According to Fahy and Rall (2007) the lower the concentration of cryoprotectant, the faster cooling must proceed to avoid ice formation Similarly, if there is an increase in viscosity or or cooling rate or decreasing the volume will increase the chances of obtaining the vitreous state (Arav et al., 2002).

Thus, the addition of cryoprotective agents (CPAs) as carbohydrates and other compounds, to the cryopreservation media increases the viscosity of the solution, thereby facilitating the vitrification process. CPAs are classified as intracellular or permeable (P-CPA) and extracellular or non-permeable (N-CPA). P-CPA are known to protect cells from cryoinjury by increasing the fluidity of the plasma membrane, lipid reordering, and partial dehydration of the cell, reducing the freezing point, thus limiting the formation of intracellular ice crystals, which is one of the main biophysical mechanisms of sperm death (Holt, 2000; Swain and Smith, 2010).

N-CPA can induce an increase in the osmolarity of the external environment, inducing the passage of water from the interior of the cell to the extracellular environment and preventing the formation of ice crystals during freezing (Meryman, 1971; Amann and Pickett, 1987; Aisen et al., 2002). N-CPA are represented by macromolecules with high molecular weight, including complex carbohydrates, such as trehalose, sucrose, and raffinose, in addition to lipoproteins from egg yolk and coconut water (Nunes, 2002), milk proteins, and some amino acids (Amann and Pickett, 1987). Osmolarity, in turn, can be divided into osmotically active molecules, such as disaccharides (sucrose and trehalose), and osmotically inactive compounds, including polysaccharides, such as maltodextrin, and proteins, such as albumin (Sieme et al., 2016).

The addition of N-CPA in the equilibrium vitrification can reduce the concentrations of permeable cryoprotectants in the vitrification solution, thereby minimizing damage from their toxicity (Shaw et al., 1997; Silva et al., 2015; Mosca et al., 2016). A study involving equine spermatozoa evaluated the use of different sugars in equilibrium vitrification; better rates of progressive motility and plasma membrane integrity were obtained with 100 mM trehalose (41.5 and 81.1% respectively) in comparison to 200 mM sucrose (28.7 and 74.4%) and 100 mM (28.6 and 72.9%). The authors attribute these results to the ability of trehalose to preserve the lipid bilayer by stabilizing the water structure around the plasma membrane, thereby protecting sperm against cryoinjuries (Consuegra et al., 2019).

Sucrose is often used in equilibrium and kinetic vitrification solutions used to increase the cell protective effect (Rall, 1987); it acts as an osmotic buffer against cellular stress caused during vitrification and devitrification (Pan et al., 2017). However, despite these benefits, N-CPA have some disadvantages in kinetic sperm vitrification. When only disaccharides are used as cryoprotectants, there is a marked reduction in motility (Table 1), whose cause remains unknown. Conversely, changes in motility during the vitrification-devitrification process may be due to changes in mitochondrial membrane potential (Isachenko et al., 2019). One way to increase the viscosity of the medium and/or thinner used and to protect the sperm plasma membrane during the kinetic vitrification process, is to include additives, such as bovine serum albumin (BSA). Table 1 shows examples of this inclusion, such as a study on rabbit sperm, in which an increase in DNA motility and integrity was observed when 0.5% BSA was combined with sucrose (0.1 M and 0.25 M) (Rosato and Iaffaldano, 2013).

**Table 1:** Studies for animal and human spermatozoa kinetic vitrification.

**Samples**	**Sample details**	**Filling process**	**Vitrification procedure**	**Results**	**References**
Frog (Unspecified specie)	Semen (*n* = unspecified)	Spermatozoa mounted on a mica film	Sucrose used. Spermatozoa immersed in liquid air for 10 seconds. Warmed in pond water at +20 °C a.	Vitrification with 1 M sucrose resulted in sperm motility recovery of 20%. At concentrations between 1 M and 2 M, intermediate results were obtained.	Luyet and Hodapp (1938)
Chicken (*Gallus domesticus*)	Semen pools (*n* = unspecified)	Spermatozoa placed in a test tube	Fructose 0.75 M used. Spermatozoa were placed in a test tube and subjected to quick frozen at -76 °C. Warmed at 42 to 45 °C^a^.	30% of spermatozoa resumed motion. No fertile eggs have been produced after artificial insemination.	Shaffner et al. (1941)
Rabbit (Unspecified specie)	Spermatozoa from vas deferens (*n*=31) and ejaculated semen samples (*n*=8)	Suspension of sperm smeared on cellophane	With or without Ringer solution. Suspension of sperm was smeared on cellophane and partially dried in air before immersing in liquid nitrogen. Warmed in 37 °C medium ^a^.	Recoverable of 0.5% motile sperm on untreated and partially dried suspension of sperm. Semen treated with hypertonic Ringer solution gave a recoverable yield of 0.1%	Hoagland and Pincus (1942)
Human	Semen (*n* = 30)	Samples of spermatozoa were located onto copper loop or into 0,25 mL straw	Cryoprotectant free. Fresh and swim-up samples used. 20 μl of sample onto copper loop or in 0.25 mL straw. Plunged into LN_2_. Warmed in 37 °C medium, 5–10 minutes.	Swim-up prepared spermatozoa without cryoprotectant onto cooper loops resulted in highest motility (49.5%) and the highest normal morphology spermatozoa when compared with other vitrification groups.	Nawroth et al. (2002)
Human	Semen (*n* = 18)	Samples of spermatozoa were located onto copper loop	Cryoprotectant free. Swim-up samples used. 20 μl of sample onto copper loop. Plunged into LN_2_. Warmed in 37 °C medium, 5–10 minutes.	Swim-up prepared spermatozoa without cryoprotectant onto cooper loops resulted in highest motility (51.5%). No significant differences in the DNA integrity of prepared spermatozoa related to presence of a cryoprotectant	Isachenko et al. (2004a)
Human	Semen (*n* = 38)	Samples of spermatozoa were located onto copper loop	Cryoprotectant free. Swim-up samples used. 20 μl plunged into LN_2_. Warmed in 37 °C medium, under intense agitation, 5–10 minutes.	40% reduction of motility of spermatozoa in comparison with swim-up-treated control.	Isachenko et al. (2004b)
				DNA integrity of cryopreserved spermatozoa was found to be unaffected.	
Human	Semen (*n* = 23)	Sperm suspension dropped into LN_2_ to form spheres	0.25M sucrose, HSA 1% and HTFb used. Swim-up samples used. 30 μl aliquots of spermatozoa suspension were dropped directly into the LN_2_, a sphere immediately forms and floats to the surface. Warmed in 37 °C medium, accompanied by gentle vortexing for 5–10 seconds.	The number of progressively motile spermatozoa was significantly higher in the sucrose-supplemented medium group (57.1± 3.2%) when compared with controls (19.4±1.9%). Supplementation of HSA and sucrose (65.2±2.6%) has a stronger cryoprotective effect on the integrity of mitochondrial membrane potential compared with HSA alone (32.6± 4.7%).	Isachenko et al. (2008)
Channel catfish (*Ictalurus punctatus*)	Semen (*n* = 4)	Sperm loaded into straws and nichrome loops	Cryoprotectant-free vitrification. 2 vitrification devices: 20µl of sperm suspension were loaded into the cut end of five straws and 15µl of sperm on nichrome loops. Plunged into LN_2_. Warmed in a water bath at 40 °C^a^.	Some twitching and vibration of sperm was observed after thawing, but no true progressive post-devitrification motility was observed. Cryoprotectant-free vitrification in nichrome loops did not yield fertilization, and in cut standard straws yielded low levels (<2%) of fertilization.	Cuevas-Uribe et al. (2011)
Rainbow trout (*Oncorhynchus mykiss*)	Semen (*n* = 10)	Sperm suspension dropped into LN_2_ to form spheres	Sucrose 0.125M, BSA 1% and 40% seminal plasma used. 20 µl of sperm suspension was dropped directly into LN_2._ Warmed in 37 °C medium with intense agitation 5-10 min.	Vitrification using sucrose 0.125M, BSA 1% and seminal plasma resulted in higher motility (82%) than other treatment groups. No cytoplasmic membrane integrity difference was found between groups. Mitochondrial membrane potentials of spermatozoa in all groups were decreased significantly comparatively with non-treated spermatozoa.	Merino et al. (2011)
Dog (German Shepherd, Golden Retriever, Labrador Retriever and Rottweiler)	Semen (*n* = 24)	Sperm suspension dropped into LN_2_ to form spheres	Sucrose (0.1, 0.25 and 0.4 M) and HTF–BSA^b^ 1% used. Swim-up samplesc. 30 µl of sperm suspension were dropped directly into LN2. Post-thaw sperm suspension was maintained at 37 °C/5% CO_2_ for 10 min and was then centrifuged at 300 g for 5 minutes.	Vitrification resulted in higher progressive motility, increased integrity of the mitochondrial membrane potential and decrease DNA fragmentation of the sperm by the addition of 0.25M sucrose when compared with sperm vitrified only with HTF medium.	Sánchez et al. (2011)
Human	Semen (*n* = 68 oligoasthenoteratozoospermic samples)	Capillary filled with sperm suspension and inserted into 0,25 mL straws	0.5M sucrose, HSA 1% and HTF used. Swim-up samples used. 50 mL plastic capillaries were manufactured from hydrophobic material. The capillary was filled with 10 μl of spermatozoa suspension by aspiration. After aspiration, the capillary was inserted into a 0.25 mL straw and plunged into LN_2_. Warmed in 37 °C medium for 20 seconds.	Vitrification in the absence of permeable cryoprotectants when compared with slow conventional freezing resulted in higher levels of motility (28.0 vs 18.0 respectively), membrane integrity (56.0 vs 22.0%, respectively) and acrossomal integrity (55% vs 21%).	Isachenko et al. (2012a)
Human	Semen (*n* = 1)	Spermatozoa suspension deposited on the end of cut standard straws (CSS)	0.5M sucrose, HSA 1% and HTF used. Swim-up samples used. A 10 μl aliquot of spermatozoa suspension was deposited on the end of the inner part of the cut standard straws (CSS) and plunged into LN_2_. Warmed in 37 °C medium in a 2-mL tube fast warming (~30 000 °C min^-1^) ^a.^.	Vitrified spermatozoa resulted in 60% progressive motility (*vs* 90% in freshly spermatozoa). 63% of spermatozoa were classified as having high mitochondrial membrane potential (*vs* 96% of freshly prepared spermatozoa).	Isachenko et al. (2012b)
Rabbit (Hybrid rabbit buck and commercial line)	Semen (*n* = 216)	Sperm suspension dropped into LN_2_ to form spheres	Cryoprotectant free or added sucrose or trehalose (0, 0.05, 0.1, or 0.25 M) with 0,5% BSA. 5 replicates of pooled sperm were immediately vitrified by dropping 30 mL semen aliquots directly in a LN_2_ bath to form frozen spheres. Warmed in a water bath at 38 °C accompanied by gentle vortexing for 10-12 seconds.	Cryoprotectant-free vitrification resulted in the null or low post devitrification recovery of motile (0%–1%) or membrane intact sperm (1%–5%), whereas DNA integrity ranged from 93% to 97%. Vitrification with BSA alone or with BSA/sucrose (0.1/0.25 M) or BSA/ trehalose (0.25 M) resulted in higher numbers of motile and membrane-intact cells.	Rosato and Iaffaldano (2013)
Human	Semen (*n* = 10)	Sperm suspension dropped into LN_2_ to form spheres	0.5M sucrose and 10mg/ml of HSA used. Swim-up samples used. 30 μl directly dropped into LN_2_ (spheres). Warmed in 42 °C medium for 10 seconds.	Vitrified spermatozoa resulted in 74.7% of total motility (vs 94.3% in freshly spermatozoa), progressive motility was 68% and membrane viability of spermatozoa was 77.21%.	Dupesh et al. (2019)
Donkey (Andalusian donkey)	Semen (n=4)	Sperm suspension dropped into LN_2_ to form spheres	0.1 M, 0.2 M and 0.3 M sucrose or 1%, 5% and 10% BSA. 30 μl directly dropped into LN_2_ (spheres). Warmed in 42 °C medium extender	Sucrose 0.1M result highest total motility and progressive motility (21.67% vs 13.42) when compared to other sucrose concentrations. The addition of different concentrations of BSA to the vitrification extender resulted in no significant differences (*p* > 0.05) in any of the sperm parameters assessed	Hidalgo et al. (2020)

^a^ Exposure time unspecified; ^b^ HTF-Human tubular fluid; BSA-Bovine Serum Albumin and HSA-Human Serum Albumin; ^c^ Swim-up method is useful in selecting motile spermatozoa as it is based on the ability of sperm to swim into the culture medium. This method may be performed by layering the culture medium directly over the semen, or layering the culture medium over the pellet, which is obtained after the centrifugation of the sample (WHO, 2010).

Another factor that has been somewhat neglected is the water content present in the sperm cell and the differences in water permeability. Differences in water permeability account largely for the magnitude of difference in optimal cooling rates for different cell types (Gao and Critser, 2000). Cryopreservation protocols must strike an equilibrium between optimal conditions for each cell type, depending on the water content, cell size and morphology, and the water permeability coefficient of the plasma membrane (Curry et al., 1994; Paoli et al., 2014). In practice, as water volume and sperm morphology are different between specie, cryopreservation protocols must also be different. Katkov et al. (2012) collected semen from three species of birds of prey (gyrfalcon - *Falco rusticolus*, golden eagle - *Aquila chrysaetos*, and eastern imperial eagle - *Aquila heliaca*) and subjected the samples to slow freezing and kinetic vitrification using the same freezing rate used for mammalian sperm. The kinetic vitrification protocol was not successful, and the authors attributed this result to morphological peculiarities and differences in the water content of bird and mammal sperm cells. For these authors, the kinetic vitrification of avian semen would require a faster freezing rate.

Another important point for obtaining the vitreous state is the volume of cryopreserved sample. According to Isachenko et al (2017) there are two methods of cryopreserving samples according to volume and contamination protection: non-aseptic methods, i.e. the one in which the sample is placed directly into liquid nitrogen, and aseptic methods in which devices are used to prevent the samples from being in direct contact with the liquid nitrogen.

Different aseptic vitrification techniques were investigated by Isachenko et al. (2005); however, only small volumes, ranging between 1 and 40 µl of sperm suspension could be vitrified in these systems and according to these authors the open-pulled straw method of vitrification is preferable because it allows isolation of the spermatozoa from liquid nitrogen, with a maximum reduction of the potential risk of microbial contamination. In 2011, the Isachenko group reported a novel aseptic cryoprotectant-free vitrification method allowing for the vitrification of larger volumes (up to 500 µl) of spermatozoa. In this study, spermatozoa vitrified with aseptic cryoprotectant-free technology displayed superior functional characteristics. The motility rate, integrity rates of cytoplasmic, and acrosomal membranes were significantly higher after vitrification than after conventional freezing (76% vs 52%, 54% vs 28% and 44% vs 30%, respectively).

## Kinetic vitrification: applications and methodologies

### Main techniques and protocols used

Kinetic vitrification, in addition to being P-CPA free, differs from other cryopreservation methods in terms of its extremely high rates of cooling (10^4^–10^6^ °C/min) and almost instantaneous devitrification, conditions that prevent the formation of ice inside the cells (Nawroth et al., 2002; Isachenko et al., 2004a). Moreover, the entire vitrification and devitrification process takes only a few seconds (Isachenko et al., 2008).

As for the use of non-permeable cryoprotectants only, Isachenko et al. (2004a) evaluated different combinations of carbohydrates (sucrose and trehalose) and proteins (human serum albumin) in men and found that slow freezing and kinetic vitrification were similar in terms of sperm motility and DNA integrity. However, in dogs, the results of kinetic vitrification using a thinner and an egg yolk medium, were not considered acceptable for the same parameters (Kim et al., 2012).

In studies using human (Isachenko et al., 2004a, b, 2005, 2008, 2011) and fish sperm (Merino et al., 2011), sperm samples were subjected to swim-up separation and centrifugation (with velocities of 300 *g* to 400 *g* for 5-10 min) prior to kinetic vitrification and after devitrification (Nawroth et al., 2002; Isachenko et al., 2004a, b, 2008). Such procedures allowed the selection of sperm with progressive motility, normal morphology, or with undamaged DNA (Isachenko et al., 2004a). This pre-selection resulted in improved sperm quality after devitrification in terms of DNA integrity (Tomlinson et al., 2001; O'Connell et al., 2003), morphology (Xue et al., 2014), and motility (Fácio et al., 2016). However, pre-processing the samples is not indicated for all species owing to the sensitivity and peculiarities that the sperm cell can present. In mice, for example, a reduction in progressive motility, mean trajectory velocity, and overall velocity after centrifugation has been observed (Shi et al., 2016).

Another peculiarity is the volume used in this technique. The most common procedure is using small sperm volumes ranging from 10 μL to 30 µL (Sanchéz et al. 2011; Slabbert et al., 2015). The great obstacle in using larger volumes was to find an aseptic method that was adequate for this increase. In 2011, the group led by Isachenko reported a new method of aseptic vitrification without the use of a cryoprotectant in humans, allowing the vitrification of larger volumes (up to 500 µL). Later, Slabbert et al. (2015) used 300 µL for human sperm vitrification and observed greater mitochondrial potential and less DNA fragmentation when compared to slow freezing.

The methods previously proposed were described as open and non-aseptic systems because the devices did not prevent the direct contact of the sample with liquid nitrogen (Isachenko et al., 2003, 2004a, 2005). Open systems were used to eliminate ice and create a glassy state in its place, requiring small cool liquid suspensions or just water at ultra-fast freezing speeds (Luyet, 1937a); the technique in which small aliquots or drops of semen (approximately 30 µL) were placed directly in liquid nitrogen aimed at achieving the desired vitrification (Isachenko et al., 2008).

Using sample isolation as an indicator of asepsis (Isachenko et al., 2017) and kinetic vitrification efficacy, different devices such as cryoloop, open pulled straw (OPS), and cut standard straw (CSS) were evaluated (Isachenko et al., 2005). In a study on human sperm, the OPS device was recommended as it allows the isolation of sperm from liquid nitrogen, with a low risk of microbial contamination during freezing and storage.

Cuevas-Uribe et al. (2011) analyzed the use of eight different devices for the vitrification of fish sperm according to parameters such as: sample filling efficiency, sample storage, sample volume, speed of cooling and heating, visualization of glass formation (characteristic of the glassy state), sample labeling, and cost per sample. According to these authors, the devices that met these parameters were the nichrome loop and the 0.25-mL CSS. Although there is no consensus on the best device, asepsis and isolation of liquid nitrogen are considered crucial for the effectiveness of the technique (Isachenko et al., 2017).

The solution used in kinetic vitrification usually consists of a thinner, an N-CPA, which can be a disaccharide, such as sucrose or trehalose, and proteins, such as human serum albumin (Slabbert et al., 2015), bovine albumin, soy lecithin (Swanson et al., 2017), fetal bovine serum, and fallopian tube fluid (Isachenko et al., 2008).

The heating of the sample during devitrification depends on the device used with varying time and temperature. Water bath or devitrification solution temperatures range from 37 °C to 42 °C, with 37 °C being reported for human, fish, dog, and domestic cat sperm (Nawroth et al., 2002; Isachenko et al., 2004a, 2004b, 2005, 2008, 2012; Merino et al., 2011; Sánchez et al., 2011; Swanson et al., 2017), 38 °C used for heating rabbit semen samples (Rosato and Iaffaldano, 2013), and 42 °C also for human semen samples (Slabbert et al., 2015).

### Main results of kinetic vitrification: animal and human models

Kinetic vitrification, as most reproductive techniques, was first tested in animal models. The first time this technique was investigated was in 1938, when Luyet and Hodapp reported the recovery of motility in 20% of sperm from frogs directly immersed in liquid nitrogen using 1 M sucrose. Later, other authors published their experiences with kinetic vitrification. In birds, Shaffner et al. (1941) subjected rooster semen to what they termed “fast freezing at -76 °C,” using different concentrations of fructose and thawing at 42 °C to 45 °C. Despite the importance of this study, as it is considered as one of the first attempts to apply kinetic vitrification to avian semen, 30% of sperm were mobile after devitrification and there were no fertile eggs after artificial insemination. In a study on rabbits, Hoagland and Pincus (1942) tried to recover sperm motility after direct vitrification in liquid nitrogen at -196 °C and obtained a recovery of 0.5% for semen without additives and 0.1% for semen diluted in Ringer’s solution. According to Katkov et al. (2012) these first efforts to perform sperm vitrification did not receive the recognition they deserved, hampered by low repeatability and survival, as well as communication difficulties due to several “iron walls” between scientists in the western allies, Germany, and the USSR, during World War II followed by the Cold War. After the contradictory results obtained by these first studies on sperm kinetic vitrification, the cryoprotective function of glycerol was discovered by Polge et al. (1949) and Smith and Polge (1950), the egg yolk was discovered by Phillips and Lardy (1940), in addition to the discovery of other CPAs, which moved the focus of the cryopreservation field from kinetic vitrification to slow (or equilibrium) freezing. Slow freezing is still the main sperm cryopreservation method (Katkov et al., 2012) in humans (Mocé et al., 2016) and domestic animals (Thananurak et al., 2019).

In the 2000s, a second “wave” of studies focused on kinetic vitrification gained momentum, pushed by the results of Nawroth et al. (2002), who vitrified human sperm without using cryoprotectants and using the swim-up technique for sperm selection, obtaining an increase in motility when compared to slow freezing with permeable cryoprotectants. Subsequently, several studies were conducted comparing vitrification with slow freezing (Isachenko et al., 2004a, 2004b), assessing different techniques and devices (Isachenko et al., 2005, 2011), and analyzing parameters beyond sperm motility (Isachenko et al., 2008, 2012). According to O’Neill et al. (2019) a major limitation to clinical implementation of vitrification for human sperm is the right balance between the volume of spermatozoa suspension cryopreserved and a standardized use of CPAs for survival of spermatozoa.

Following a line of studies similar to that used by the group led by Isachenko, some researchers have evaluated the application of kinetic vitrification to the semen of dogs, fish, and rabbits (Table 1). The results of these studies differ depending on the species, kinetic vitrification protocol and evaluated semen quality parameters. In fish, for example, two studies were conducted in completely different ways: in the first study (Merino et al., 2011) used sucrose as N-CPA and bovine serum albumin (BSA) and seminal plasma as additives. The sample was previously centrifuged and placed directly into liquid nitrogen; while the second study (Cuevas-Uribe et al., 2011) does not use any type of cryoprotector or additives and compares two devices the cut straw and the nichrome loop. In the first experiment motility was obtained above 80% and plasma membrane integrity for the BSA + seminal plasma combination was 86.7%. In the second, no motility was obtained and the parameter evaluated was fertility which was also low (<2%).

As previously mentioned, the water volume and sperm cell morphology of different species are different, and this may be one of the factors why it is not possible to replicate the same kinetic vitrification protocol in all species. In addition, it is known for example that cryopreservation affects plasma membrane integrity and that plasma membrane composition varies among species (Gautier and Aurich, 2022) therefore some species are more susceptible to damage during and after cryopreservation. For kinetic vitrification, more studies on these aspects are still needed to make a more precise statement about the interference of the protocol on the quality of the devitrified semen.

This demonstrates that it is necessary that the same protocol and evaluations for the quality of devitrified semen be applied more than once in experiments with similar animal groups or the same species to obtain accuracy of results and repeatability. To date, this is observed in experiments using human spermatozoa, where the number of publications is higher than for animal species.

## Final considerations

Kinetic vitrification has great potential as any emerging technology and has recently become an alternative technique showing encouraging results for the use of vitrified sperm in assisted reproductive technologies such as in vitro fertilization (IVF) or intracytoplasmatic sperm injection (ICSI) (Isachenko et al., 2012; Nagashima et al., 2015; Cerdeira et al., 2020).

The advantages of this methodology include its simplicity, speed, low cost, and the preservation of important physiological parameters, such as mitochondrial membrane potential and DNA integrity (Sánchez et al., 2011). In addition, cryoprotectant-free vitrification can induce less biological changes in human spermatozoa, in comparison with conventional freezing (Wang et al., 2022) and lower acrossomal changes in dog spermatozoa (Caturla-Sánchez et al., 2018). Another advantage is that the use of high concentrations of permeable cryoprotectants, as used in equilibrium vitrification, are not necessary for kinetic vitrification (O'Neill et al., 2019) and it can reduce damage effects to sperm cell.

Also, cryopreservation success is measured by the motility after devitrification, and kinetic vitrification has not yet achieved satisfactory results in this regard in studies for some animals. In dogs, for example, acceptable data were not obtained after devitrification using only sucrose as a non-permeable cryoprotectant requiring the inclusion of proteins, such as bovine serum albumin (Sánchez et al., 2011) as an additive to stabilize the sperm membrane. Therefore, further studies should investigate which additives can stimulate sperm kinetics after devitrification and what are the heating rates specific for each species. The optimal concentration of non-permeable CPAs is a key factor for sperm vitrification success. It is species-specific but also depends on the methodology (Hidalgo, 2021) and need additional attention in terms of research.
